# Case of Stricture of the Ileum, and Perforation of the Appendix Vermiformis Cæci

**Published:** 1844-04-06

**Authors:** 


### THE MEDICAL EXAMINER,

### And Record of Medical Science.

Vol. VII.] PHILADELPHIA, SATURDAY, APRIL 6, 1844. [No. 7.

PHILADELPHIA, March 28, 1844,

109 S. Tenth street.

CASE OF STRICTURE OF THE ILEUM, AND PERFORATION OF THE APPENDIX VERMIFORMIS CÆCI.

MY DEAR SIR:—The following notes of the last illness, and morbid appearances on the dissection, of one who had just attained those honours which would have enabled him to commence a career of professional usefulness and distinction, have been drawn up by the friend of the deceased—Dr. Child—at whose house he was attacked, whilst on a visit, with the malady that proved fatal, and whose attention to his friend was most devoted. Dr. Southwick was from Vassalboro', Maine, and had attended the medical lectures in Philadelphia for two sessions. He had passed an examination, most honourable to himself, in Jefferson Medical College; and, two days before the Commencement, when the degree was to have been conferred upon him in person, experienced the first symptoms of his malady. His first course of lectures he attended partly in Jefferson Medical College, and partly in the University of Pennsylvania. His last course was wholly in the former Institution. In both sessions, however, he attended the department of Institutes of Medicine—to the study of which he was much attached—in the two Schools. He had paid much attention to Chemistry, and his thesis, which was highly thought of by Professor Bache, as well as by myself, to whom it was submitted, was "On the Relations of Chemistry to Pathology." Histology, healthy and morbid, was arduously pursued by him, and he was a zealous and accurate microscopist. Some of the results of his histological examinations have appeared in the "Examiner." By those with whom he was personally acquainted in both Institutions, his zeal and acquirements were highly appreciated; and all must deplore, that a career, so auspiciously commenced, should have been thus prematurely terminated. But I need not say more to you, who knew him so well, and so favourably.

Believe me, my dear sir,
Very truly yours,
ROBLEY DUNGLISON.

Professor R. M. Huston.

---

PHILADELPHIA, 3d mo. 29th, 1844.

To Professor Dunglison.

DEAR DOCTOR:—The following are the notes of the case of our late lamented friend, Dr. Edward W. Southwick.

On the night of the 18th inst. he had a severe attack of bilious colic, with the usual symptoms of pain, flatulence, and bilious vomiting. For this he had warm fomentations over the abdomen—Opii Acetat. gtt. xxv.—every two hours, and warm enemata. On the morning of the 19th, the pain and vomiting had abated; and there was some meteorism, with slight tenderness of the abdomen on pressure. In the afternoon, I gave a Seidlitz powder, and in the evening, half a fluidounce of Ol. Ricini. The pulse was natural, but no evacuation from the bowels: the enemata were therefore repeated. On the 20th, in the morning, the castor oil was again given, with 25 drops of the Oleum Terebinthinæ. In the afternoon, Dr. Dunglison was requested to see him with me; the pulse was still normal; there had been no evacuation since the morning of the 18th inst.; there was but little soreness of the abdomen, which was somewhat tympanitic, except over the region of the cœcum, where it was dull on percussion. Professor Dunglison diagnosticated subacute peritonitis, with obstruction of the cœcum; expressed his fears as to the result of the case; directed a continuation of the soothing course of treatment; and suggested the following pill:

℞. Hydrarg. chlor. mit., gr. x.
Pulv. opii, gr. iiss. M.

To be taken immediately: also, an enema, with Ol. Ricini, f.℥iss. and if no evacuation occurred by morning, an enema, with f.ʒiij. of Oleum Terebinthinæ, and the application of twenty leeches to the abdomen, to be followed by warm cataplasms.

On the 21st, there had still been no evacuation; but the tenderness and meteorism had greatly diminished. Dr. Dunglison directed twenty-five leeches over the region of the cœcum, the continuation of the fomentations, and the following mixture:

℞. Ol. ricini, f.℥iss.
Aq. menth., f.℥iiiss.
Mucilag. acac., f.℥iss. M.

Two table-spoonsful to be taken every two hours until the bowels are opened.

In the evening, he had a warm bath and a large enema of warm water. At two o'clock on the morning of the 22d, he had a profuse fluid evacuation, containing fœcal matter; after which he was much prostrated. He now took some light nourishment, vomited several times through the day, and had thirteen or fourteen evacuations in the 24 hours. The soreness of the abdomen was almost gone, yet there remained great dulness over the region of the cœcum: he could move without much pain, but complained of a "*death-like sickness*," for which he took ice water, soda water, opiates, and Spt. Æther. Nit. at proper intervals, but without any relief. At two o'clock on the morning of the 23d, he vomited a large quantity of fluid matter, of the colour, consistence, and smell of the evacuations from the bowels. At six o'clock, he had a second attack of stercoraceous vomiting. On the morning of the 23d, Dr. Dunglison directed a quarter of a drop of creosote in camphor water, to allay the sickness, and a sinapism to the epigastric region; but the sickness continued. In the afternoon, Professor Meigs saw him. The abdomen was about in the same condition as in the morning; the dulness on percussion was found to extend along the ascending colon; he was very restless, but had not much pain; the death-like sickness, too, had somewhat abated, but he complained of uneasiness in the region of the heart. During the night, he took gtt. 150 of McMunns' Elix. Opii, and one grain of solid opium; but got no rest. He complained of a sharp, pungent pain in the bladder, as he supposed. At 3, A. M., on the 24th, I observed his pulse to be accelerated for the first time. It had not exceeded 80, nor had the skin been morbidly hot; but the pulse now beat 125 in the minute. He had vomited but once in the night; the ejected matter being chiefly bile and the gastric contents. At 6, A. M., the pulse was 150 per minute. Throughout the disease, the countenance had been anxious.

At ten o'clock, when Drs. Dunglison and Meigs saw him, the pulse was 160, and the restlessness had increased, so as to cause him to be constantly moving about the bed. The pain of the abdomen on pressure was slight. He readily permitted his medical attendants to percuss the whole abdomen, and even to make considerable pressure. The dulness over the cœcum and ascending colon was as marked as ever. His most distressing feeling was an indescribable anxiety, which he referred to the epigastrium. It was now plain that he was sinking, and that he probably would not live many hours. At 12, M., he lay very quietly, the restlessness having given place to a calm, which was the precursor of rapid dissolution. At one, the pulse was imperceptible at the wrist; and at twenty-five minutes before 4, P. M., he died.

Twenty-four hours after dissolution, the body was examined by me in the presence of Drs. Dunglison, Meigs, Pancoast, North, Bibighaus, Griffin, Piper, and Robie. We found the whole abdomen tympanitic—the dulness, which had been so marked over the region of the cœcum, having disappeared. On opening the cavity of the peritoneum, a portion of fluid fœcal matter was found: very extensive recent adhesions existed between the folds of the intestines, and also between the intestines and the abdominal parietes, particularly on the left side, which had been tympanitic. The peritoneum was generally injected, and, in certain parts, very much so. There was a large quantity of fibrinous matter thrown out, which had formed bands in various directions over the intestines. The greatest evidences of injection and inflammation were around the cœcum and the ascending colon; there was a firm stricture over the ileum, about an inch from its termination in the cœcum, which bound it down to the right side of the body of one of the vertebræ. In the appendix vermiformis cœci, there was a perforation sufficiently large to admit the handle of a scalpel, when passed from the intestine; this had allowed fœcal matter to escape into the abdomen. A small mass of hardened fæces was in the cavity of the abdomen, about three lines long and one in diameter, which had probably been lodged in the appendix.

In this brief and hasty account, I have omitted saying anything in relation to the character of the deceased. The views expressed in the resolutions of a meeting of his former classmates, at which I had the honor to preside, are but a feeble tribute to the worth of our estimable young friend, whose loss we all so deeply deplore.

With sentiments of high regard and esteem,

I remain, affectionately,

HENRY T. CHILD, M.D.

*No.* 161 *Coates street.*

BY PROFESSOR DUNGLISON.

